# Introduction of Non-natural Amino Acids Into T-Cell Epitopes to Mitigate Peptide-Specific T-Cell Responses

**DOI:** 10.3389/fimmu.2021.637963

**Published:** 2021-03-11

**Authors:** Aurélien Azam, Sergio Mallart, Stephane Illiano, Olivier Duclos, Catherine Prades, Bernard Maillère

**Affiliations:** ^1^Sanofi, Biologics Research, Vitry-sur-Seine, France; ^2^Université de Paris-Saclay, CEA, INRAE, Département Médicaments et Technologies pour la Santé, SIMoS, Gif-sur-Yvette, France; ^3^Sanofi R&D, Integrated Drug Discovery, Chilly-Mazarin, France; ^4^Sanofi R&D, Cardiovascular Diseases & Metabolism, Chilly-Mazarin, France

**Keywords:** non-natural amino acids, immunogenicity, HLA class II molecules, CD4 T-cell response, T-cell epitope

## Abstract

Non-natural modifications are widely introduced into peptides to improve their therapeutic efficacy, but their impact on immunogenicity remains largely unknown. As the CD4 T-cell response is a key factor in triggering immunogenicity, we investigated the effect of introducing D-amino acids (Daa), amino isobutyric acid (Aib), N-methylation, C_α_-methylation, reduced amide, and peptoid bonds into an immunoprevalent T-cell epitope on binding to a set of HLA-DR molecules, recognition, and priming of human T cells. Modifications are differentially accepted at multiple positions, but are all tolerated in the flanking regions. Introduction of Aib and Daa in the binding core had the most deleterious effect on binding to HLA-DR molecules and T-cell activation. Their introduction at the positions close to the P1 anchor residue abolished T-cell priming, suggesting they might be sufficient to dampen peptide immunogenicity. Other modifications led to variable effects on binding to HLA-DR molecules and T-cell reactivity, but none exhibited an increased ability to stimulate T cells. Altogether, non-natural modifications appear generally to diminish binding to HLA-DR molecules and hence T-cell stimulation. These data might guide the design of therapeutic peptides to make them less immunogenic.

## Introduction

Therapeutic peptides represent approximately one-tenth of marketed drugs, owing to their remarkable target selectivity, the high flexibility of their sequence and their low toxicity (1). As therapeutic peptides are sensitive to enzymatic proteolysis, most contain non-natural modifications whose beneficial effects on stability, structural conformation and bioavailability have been well-depicted in the literature (2, 3). The immunogenicity of therapeutic peptides is an important issue in their development and safe use in humans (4). Specific antibodies resulting from injections of therapeutic peptides might increase or decrease their pharmacokinetics, induce a therapeutic loss or provoke autoimmune and allergic symptoms (4). However, effects on immunogenicity of most the non-natural modifications introduced into therapeutic peptides have scarcely been investigated, and mostly in animal models (5–9). Immunogenicity is the result of multiple cellular and molecular processes among which the capacity to be presented by HLA class II molecules and to stimulate CD4 T cells appears as a main factor (10). After being activated, peptide-specific CD4 T cells provide appropriate cognate interactions and cytokines to promote the differentiation of B lymphocytes into antibody-producing plasma cells. Owing to the requirement for T-cell epitopes to bind to HLA class II molecules, predictive approaches have been developed to identify T-cell epitopes relying on the modeling of peptide interactions with HLA molecules (11). However, T-cell epitope prediction programs have been trained with natural sequences (12, 13) and therefore are not appropriate to evaluate the immunogenicity of peptides containing non-natural modifications. Although non-natural modifications have been widely introduced in therapeutic peptides, their impact on immunogenicity in humans cannot be anticipated.

A large library of non-natural modifications exists and some of them are widely used in medicinal chemistry. They mainly consist of D-amino acids (Daa), N-methylation (Nm), C_α_-methylation (Cm), amino isobutyric acids (Aib), Ψ(CH_2_NH) reduced amide bonds (Rd), and peptoids (Pp). Daa are the enantiomeric forms of L-amino acids. Incorporation of Daa into peptides has been associated with low immunogenicity in mice (5, 7, 8) and reduced binding to HLA-DR1 and DR4 molecules (14). Aib consists of α,α-dimethyl glycine and seems to increase peptide antigenicity (15, 16). Nm, Ψ(CH_2_NH) Rd, and Pp are modifications introduced in the peptide bond. Studies on their immunogenicity have been limited to assessing their effects on binding to MHC class II molecules (9, 14, 17) and on antibody (9) and T-cell recognition (9, 18, 19). To our knowledge, no immunological studies have reported the immunological properties of peptides carrying Cm. There is a mosaic of data on the immunogenicity of peptides containing non-natural chemical modifications, but it does not provide any clues on how chemical modifications could be introduced in a sequence to reduce immunogenicity in humans.

We therefore addressed this issue by evaluating the effects of introducing six frequent non-natural modifications into an immunogenic peptide on the ability to bind to HLA class II molecules and to activate human CD4 T cells. A wide range of T-cell reactivity and HLA binding activity was found for the 69 investigated analogs, many of which resulted in substantial loss of activity.

## Methods

### Peptides

**Figure 1:**
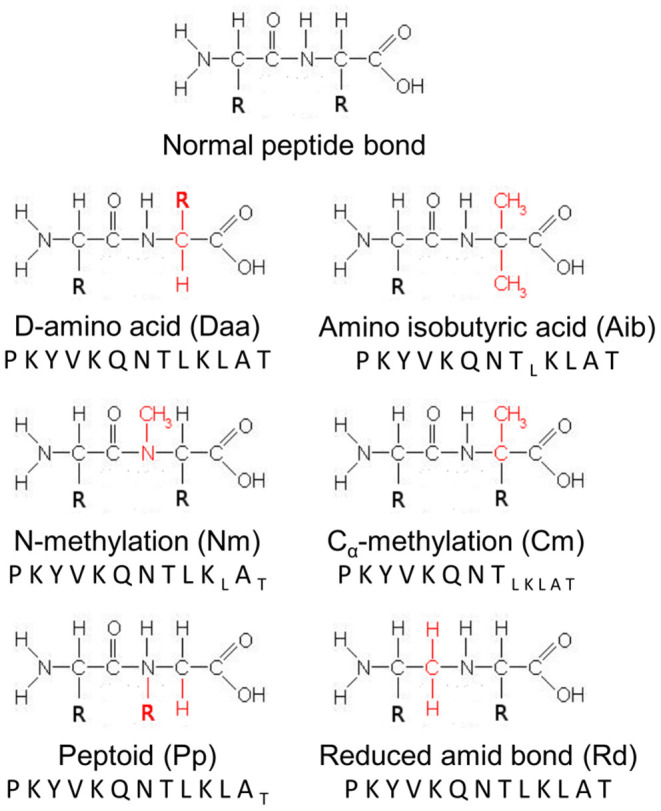
Schematic representation of modifications introduced into the HA peptide. A normal peptide bond is represented as reference. R refers to the side-chains of the amino acids involved in the peptide bond, while chemical groups colored red designate the chemical modifications introduced at each position of the HA peptide sequence. Letters in subscript refer to analogs, which could not be synthesized by the manufacturer and tested.

The influenza hemagglutinin (HA) peptide HA_306−318_ and the 69 analogs (Figure 1) were from Peptides&Elephants GmbH (Hennigsdorf, Germany).

### Human Cell Preparation and HLA-DR Genotyping

Peripheral blood mononuclear cells (PBMCs) were obtained from blood cells collected at the Etablissement Français du Sang (EFS, Rungis, France) as buffy coat preparations from anonymous healthy donors who gave informed consent, in accordance with EFS guidelines. PBMCs were isolated by Ficoll-Paque PLUS density gradient centrifugation (GE Healthcare, Buc, France). Monocyte-derived DCs were generated from plastic-adherent PBMCs after 4 or 5 days of culture in AIM-V medium (Invitrogen, Villebon-sur-Yvette, France) supplemented with 1,000 U/mL of recombinant human IL-4 (rh-IL-4; R&D systems, Lille, France) and of rh-GM-CSF (R&D systems). Monocyte-derived DCs were matured with 1 μg/mL of lipopolysaccharide (LPS, Sigma, St. Louis, MO, USA) for 2 days at 37°C. CD4 T cells were isolated from autologous PBMCs by positive selection using an anti-CD4 monoclonal antibody coupled to magnetic microbeads (Miltenyi Biotech, Paris, France) and magnetic cell sorting, as recommended by the manufacturer. The HLA-DR genotypes were determined using the Gold SSP DRB1 typing kit (Invitrogen) after DNA extraction from PBMCs with the NucleoSpin Blood L Kit (Macherey Nagel, Hoerdt, France).

### Short-Term T-Cell Culture

PBMCs were seeded at 5 M/mL with the peptide HA (10 μg/mL) in RPMI 1640 medium supplemented with 5% SAB, 10 U/mL IL-2 (R&D systems) and 1 μg/mL anti-CD28 (Miltenyi). The culture medium was changed every 2–3 days. Cells were harvested at day 10 and submitted to interferon-γ (IFN-γ) enzyme-linked immunospot (ELISPOT) assay with HA or analogs as described below. This assay was carried out with 4 different healthy donors.

### Generation of Peptide-Specific T-Cell Lines

Mature dendritic cells (mDCs) were loaded for 4 h at 37°C with either the peptide HA or analogs (3 μM). After washings, loaded DCs were added (10 replicates per peptide) at 20,000 cells/well to round-bottom microwells with 200,000 autologous CD4^+^ T cells in 200 μL of Iscove's modified Dulbecco medium supplemented by 10% human AB serum (IMDM, Lonza, Levallois-Perret, France), 1,000 U/mL rh-IL-6 (R&D systems) and 10 ng/mL rh-IL-12 (R&D systems). Co-cultures were incubated at 37°C in 5% CO_2_ for 21 days. The CD4^+^ T cells were restimulated on days 7 and 14 with fresh autologous peptide-loaded DCs, 10 U/mL IL-2 (R&D systems) and 5 ng/mL IL-7 (R&D systems). The specificity of each cell culture replicate (CD4^+^ T-cell line) was assessed by IFN-γ ELISPOT assay using HA or selected analogs at day 21. T cell lines were generated from 7 healthy donors, different from the previous donors.

### IFN-γ ELISPOT Assay

Fifty thousand cells from the short-term culture or a mix of 50,000 cells from T-cell lines and 50,000 autologous PBMCs were incubated with HA or analogs (10 μg/mL) in Multiscreen 96-well plates (Merck Millipore, Bedford, MA, USA) previously coated with 2.5 μg/mL anti-human IFN-γ monoclonal antibody (mAb, 1-D1K; Mabtech, Nacka, Sweden). After overnight incubation, spots were revealed using 0.25 μg/mL biotinylated anti-human IFN-γ mAb (7-B6-1; Mabtech) in phosphate-buffered saline/1% bovine serum albumin, extravidin-phosphatase (dilution 1:3,000 in phosphate-buffered saline/0.05% Tween 20/1% bovine serum albumin; Sigma-Aldrich, Saint-Quentin Fallavier, France), and NBT/BCIP (Sigma-Aldrich). Spot number was determined by the AID ELISPOT Reader System (AID GmbH, Ebinger, Germany). PHA was introduced in the assay as positive control. T-cell responses was considered as specific when a spot count was 2-fold higher in the presence of the peptide than in its absence, with a minimal difference of 25 spots.

### HLA-DR-Specific Binding Assays

Human leukocyte antigen-DR molecules were immunopurified from homozygous EBV B lymphoblastoid cells as previously reported (20). HLA-DR molecules were immunopurified by affinity chromatography using monomorphic mAbs L243 (American Type Culture Collection, Manassas, VA). Binding of HA and analogs to HLA-DR molecules (HLA-DRB1^*^01:01, 04:01, 07:01, 11:01, and DRB5^*^01:01) was assessed by competitive ELISA (20, 21). Binding assays were performed by diluting HLA class II molecules with the biotinylated HA and serial dilutions of HA or competitor analogs. After 24- to 72-h incubation and pH neutralization, samples were applied to 96-well ELISA plates (Nunc MaxiSorp, Invitrogen) previously coated with 10 μg/mL L243. Bound biotinylated HA was detected by addition of streptavidin-alkaline phosphatase conjugate (GE Healthcare), and 4-methylumbelliferyl phosphate substrate (Sigma-Aldrich). Emitted fluorescence was measured at 450 nm upon excitation at 365 nm. Peptide concentrations that prevented binding of 50% of the labeled peptide (IC_50_) were calculated. IC_50_ values of HA for each HLA class II molecule were 0.8 nM (DRB1^*^0101), 37 nM (DRB1^*^0401), 29 nM (DRB1^*^0701), 20 nM (DRB1^*^1101), and 8 nM (DRB5^*^0101). Data were reported as relative affinity corresponding to the ratio of the IC_50_ of the tested analog to that of HA. Means were calculated from at least two independent experiments.

## Results

### Recognition of HA Analogs by HA-Specific T Cells

**Figure 2:**
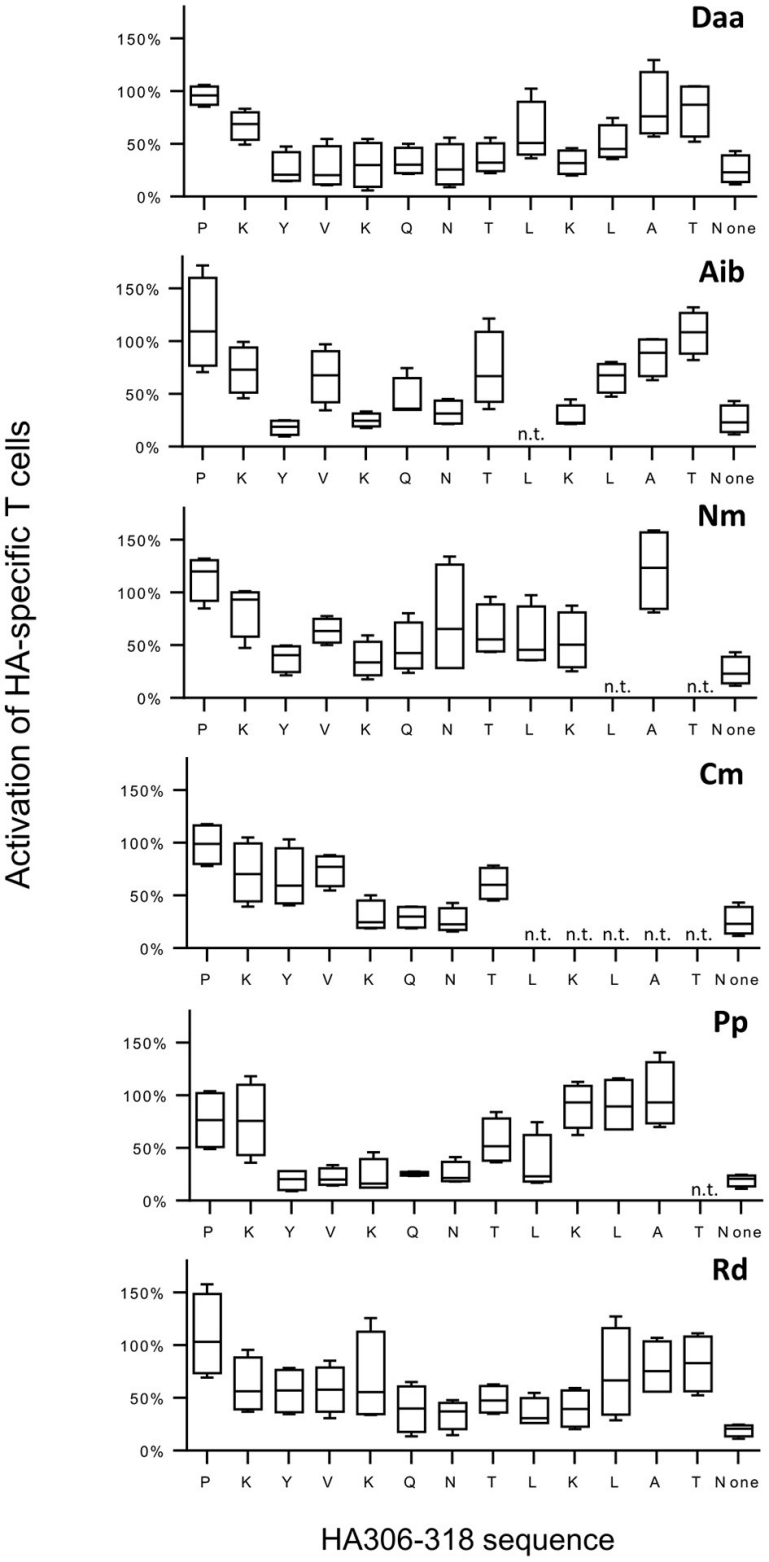
*In vitro* antigenicity of modified HA analogs toward HA-specific memory T cells. After a short-term culture of PBMCs from 4 healthy donors with HA, CD4 T cells were submitted to IFN-γ ELISPOT using HA, individual analogs or culture medium (“none” for “no peptide”). Data were reported as relative T-cell reactivity, corresponding to the ratio of the mean of IFN-γ spot numbers for the analog by the mean IFN-γ spot numbers for HA across the four healthy responding donors. Boxes extend from the 25th−75th percentiles, the line in the middle of the box is plotted at the median and the whiskers go down to the smallest value and up to the largest. n.t., not tested. The HLA-DRB1 typing of the four donors were the following: donor A07 (04:01/11:01); donor A12 (03:01/13:03); donor A13 (01:01/15:01); and donor A18 (01:01/13:01).

To evaluate the impact of non-natural modifications on T-cell response, we selected the HA_306−318_ peptide (HA) as a T-cell epitope model. HA binds to multiple HLA-DR molecules (22) and participates in the Flu-specific CD4 T-cell response in multiple donors as a result of its HLA-DR promiscuity (23). We first evaluated the recognition of 69 HA analogs by HA-specific T cells. Briefly, PBMCs from 4 donors with different HLA typing were enriched in HA-specific T cells by a short-term culture of 10 days with the HA peptide and submitted to IFN-γ ELISPOT assay with HA or each of the 69 analogs (Figure 2). HA interacts with the HLA-DR molecules by a peptide core starting at position Y_308_ and ending at position L_316_, the remaining amino acids in N and C termini of the peptide constituting the flanking regions (24, 25). As shown in Figure 2, almost all the substitutions in the flanking regions led to a limited reduction of the T-cell activation and to a slight increase by introduction of Nm at position A_317_ and of Aib at positions P_306_ and T_318._ In contrast, multiple substitutions in the core region diminished T-cell recognition. Introduction of Daa at 8 positions and of Aib at 5 positions (Y_308_, K_310_, Q_111_, N_312_, and K_315_) within the peptide core gave rise to weak T-cell activation, while substitution of Nm, Cm, Pp, and Rd at 3–6 core positions induced 50% T-cell activation. Introduction of a single non-natural amino acid into the HA peptide could therefore dramatically alter its T-cell antigenicity, depending on the nature of the modifications, and the position in the peptide sequence.

### Relative Affinity of HA Analogs for HLA Class II Molecules

**Figure 3:**
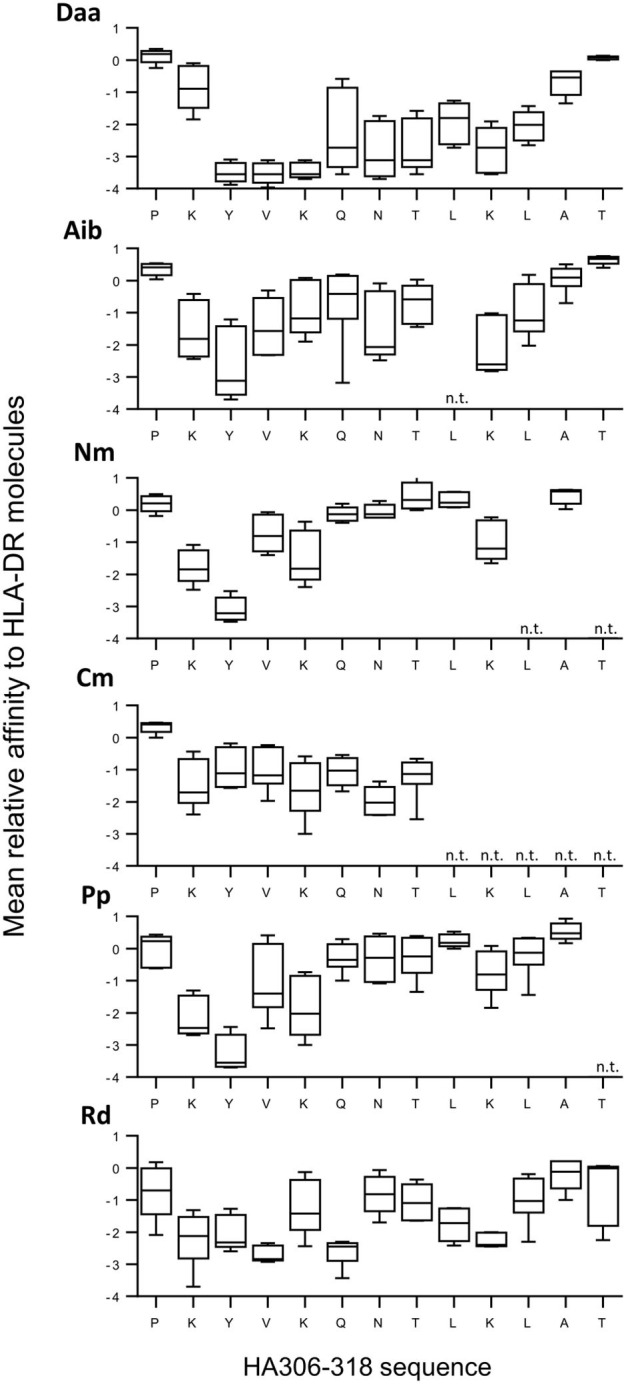
Binding of modified HA analogs to five common human leukocyte antigen (HLA)-DR molecules. HA and 69 analogs were submitted to competitive ELISA specific for the HLA-DR molecules (DRB1*01:01, 04:01, 07:01, 11:01, and DRB5*01:01). Data were expressed as relative affinity for HLA-DR molecules (mean ratio of IC_50_ of analog to the IC_50_ of the HA peptide). Boxes extend from the 25th−75th percentiles, the line in the middle of the box is plotted at the median and the whiskers go down to the smallest value and up to the largest. n.t., not tested.

**Figure 4:**
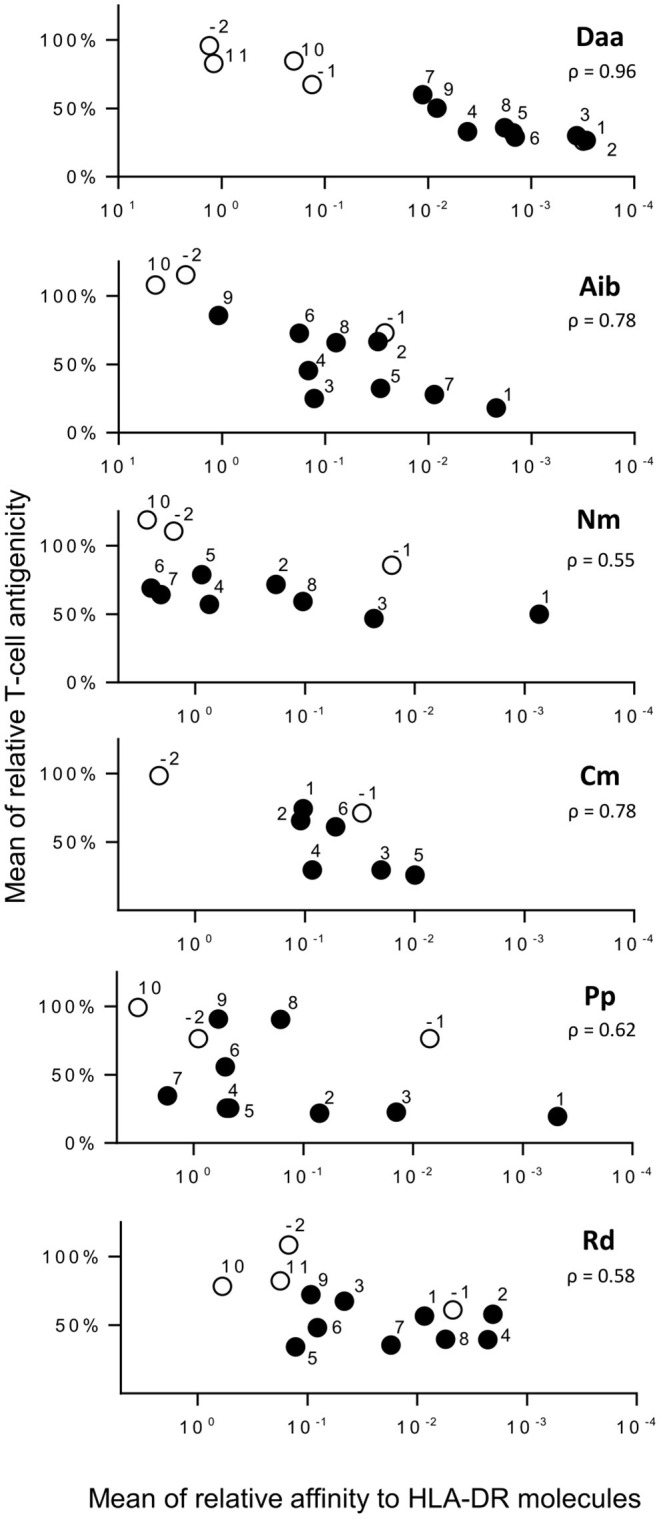
Comparison of T-cell antigenicity of HA analogs and their binding to HLA-DR molecules. Relative T-cell reactivity with HA-specific T cells of each substitution (ratio of IFN-γ spot number of each analog to that of HA) is reported as a function of their mean relative affinities for HLA-DR molecules (mean ratio of IC_50_ of analog to IC_50_ of HA across the five HLA-DR molecules). Numbers refer to the position relative to the P1 anchor residue of HA. Positions within the core or within the flanking regions are in black and white, respectively. ρ refers to Spearman's rank correlation coefficient.

Among the four responding donors, three shared the same HLA-DRB1^*^01:01 allele, while one was typed HLA-DRB1^*^04:01 and 11:01. These three allotypes are known to bind the HA peptide as well as HLA-DRB1^*^07:01 and DRB5^*^01:01 (22). We therefore assessed the binding capacity of all the HA analogs for these five molecules to evaluate the impact of non-natural modifications on the binding to HLA-DR molecules (Figure 3 and Supplementary Tables 1, 2). Substitutions of amino acids residing in the HA flanking regions were generally well-tolerated, K_307_ excepted. Introduced in the core region, Daa induced strong reducing effects, the loss of affinity at positions Y_308_, V_309_, and K_310_ being greater than a factor of 1,000. Strong effects were also observed by introducing Aib, Nm, Pp, Rd at position Y_308_. Substitution by one of these four modifications at several other positions of the peptide core gave rise to a >100-fold binding loss, but many other positions appeared permissive to non-natural modifications. Only modification Cm entailed few reducing effects. To decipher the role of the binding loss in the T-cell recognition of each substitution, we plotted T-cell reactivity with HA-specific T cells of each analog as a function of its mean relative affinity for the 5 HLA-DR molecules (Figure 4). A good correlation was found for Daa (ρ = 0.9648), Aib (ρ = 0.7802), and Cm (ρ = 0.7833), indicating that loss of T-cell recognition resulted from loss of binding to HLA-DR molecules. In contrast, the correlation was less pronounced for Nm, Pp, and Rd, as multiple substitutions affected T-cell recognition without impairing binding to HLA-DR.

### T-Cell Priming Capacity of Aib and Daa Modified Analogs

**Figure 5:**
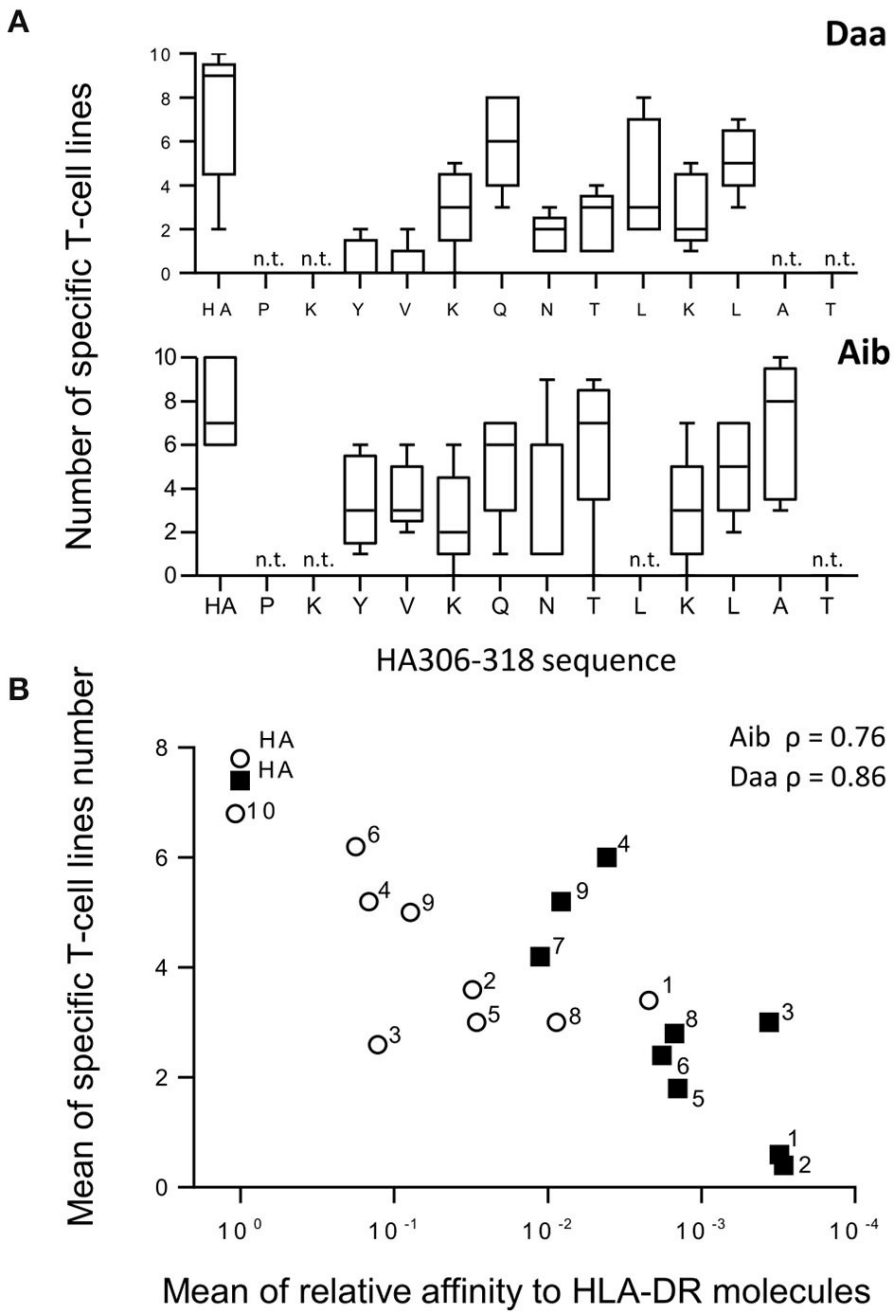
Generation of CD4 T-cell lines specific for Aib and Daa analogs. **(A)** CD4 T-cell lines were produced by 3 weekly rounds of stimulation with autologous mature DCs previously loaded with either HA or each analog using cells collected from 5 different donors. Specific T-cell lines were identified by IFN-γ ELISPOT from 10 independent cell cultures per peptide, each analog being identified by the modified position in the HA sequence (x axis). Boxes extend from the 25th−75th percentiles, the line in the middle of the box is plotted at the median and the whiskers go down to the smallest value and up to the largest. n.t., not tested. **(B)** Mean number of T-cell lines specific for Aib (open circle) and Daa analogs (closed square) was plotted as a function of their HLA-DR binding (ratio of IC_50_ of analog to that of HA). Numbers refer to the position relative to the P1 anchor residue of HA. ρ refers to Spearman's rank correlation coefficient.

As the Aib and Daa analogs gave rise to the most dramatic effects, we evaluated their capacity to initiate a *de novo* peptide-specific T-cell response. We therefore submitted Aid and Daa modified peptides to a long-term T-cell assay using cells collected from donors with different HLA typing. This assay has been already used to identify multiple viral epitopes from non-exposed healthy donors (26–28) and hence to detect peptide-specific naïve T cells besides memory T cells. Accordingly, of the seven donors who responded to HA in the long-term T-cell assay (Figure 5), at least three did not respond in the short-term T-cell assay, which is appropriate to detect memory cells (data not shown). As shown in Figure 5, Daa substitutions in the core sequence led to a large decrease in the ability of the analogs to prime T cells, the analogs modified at positions Y_308_ and V_309_ being almost unable to elicit CD4 T cells in this assay. Introduction of Aib also appeared to decrease the T-cell priming of the corresponding analogs, but the decrease was smaller. A good correlation (ρ = 0.86 for Daa and ρ = 0.76 for Aib) was found between T-cell priming and HLA binding, suggesting that the loss of priming activity resulted from the binding loss (Figure 5B). Finally, an at least 3-log loss of binding activity appeared to be required to abolish the T-cell priming of the analogs in this assay.

## Discussion

Because of the immunogenicity risk of therapeutic peptides, we investigated the effects of non-natural modifications introduced in a T-cell epitope model on binding to HLA class II molecules, T-cell antigenicity and T-cell priming. These experiments were performed using HLA-unrelated healthy donors and took advantage of the promiscuous binding to HLA molecules of the HA model peptide (22) and of its frequent T-cell reactivity in healthy donors (23). Multiple non-natural substitutions introduced in the peptide were found to diminish T-cell activation and hence potential immunogenicity.

In line with their loss of binding to HLA-DR molecules, multiple Daa, and Aib analogs exhibited a loss of T-cell recognition by HA-specific T cells and a reduced capacity to prime CD4 T cells in a long-term T-cell assay. Long-term T-cell assay revealed T-cell epitopes from viruses (26, 27, 29), tumor antigens (30, 31), and therapeutic antibodies (32, 33), which are recognized from the naïve repertoire, the T cell being collected from donors, who have not been exposed to antigens. We observed very good concordance with their immunogenicity in humans (32, 34), the size of the naïve T-cell repertoire being correlated with the intensity of the memory response (35, 36). Our results therefore suggest that introduction of Daa and Aib into a peptide should be followed by a reduction of immunogenicity of the modified peptides. Many studies have reported the weak immunogenicity in mice of peptides (8, 37), proteins (7), and polymers (5) that contain D-amino acids. The authors attributed this effect to the inability of the compounds to be processed by the cellular machinery, thus making them unable to be presented to T cells (5). We confirmed previous studies on HLA-DRB1^*^01:01 and DRB1^*^04:01 molecules (14, 38) and extended these observations to HLA-DRB1^*^07:01, DRB1^*^11:01 and DRB5^*^01:01, by showing that introduction of Daa within the core of the HA peptide, even in the TCR-oriented positions (P2-V_309_, P3-K_310_, P5-N_312_, and P8-K_315_), deeply affected the affinity for HLA-DR molecules. Binding to HLA-DR molecules is the result of interactions with peptide side chains, which are accommodated in five specificity pockets (P1, P4, P6, P7, and P9) and 15 hydrogen bonds between mainly conserved residues of the HLA-DR molecules and the main chain of the peptide (24, 25). This abundant network of hydrogen bonds actively helps to anchor the peptide in the binding groove of the HLA-DR molecules and to enlarge the peptide specificity of the HLA class II molecules, allowing them to display a multitude of peptides (24, 25, 39). Introduction of Daa changes the orientation of the side-chains and hence might prevent accommodation of the anchor residues into the specificity pockets. Introduction of Daa at other positions also disturbed the binding of the peptide suggesting that Daa altered the peptide conformation and affected interactions with both side chains and main chain of the peptide. As shown in Figures 4, 5, the loss of binding to HLA-DR molecules was correlated with the loss of T-cell recognition or priming and therefore appeared as the main factor responsible for the loss of T-cell activation.

To a lesser degree, Aib also reduced the binding of the modified peptides to HLA-DR molecules and their capacity to prime the T-cell response. Addition of methyl group at this position of the peptide backbone does not directly mask H-bonds with the HLA molecule. However, the two methyl groups on the alpha carbon of Aib impose major steric restrictions on the peptide conformations and seem to promote helical folding of the peptide (40). The adopted conformation may increase peptide antigenicity (15, 16), but seems poorly compatible with the extended conformation of a peptide bound to HLA class II molecules, throughout the core peptide region (24). This conformational change might therefore affect the peptide interactions with the HLA-DR molecule. Aib may also introduce steric hindrance, which reduces the binding of the peptide in the groove of the HLA class II molecules.

Other modifications we introduced were in the peptide bonds of the HA peptide and exhibited more variable impacts than Aib and Daa on binding to HLA class II molecules and T-cell recognition. Nm at multiple positions of the HA peptide had few effects on the binding to HLA class II molecules, in agreement with previous studies (9, 14, 38) and on T-cell recognition (9). Modification of the peptide backbone by Nm directly suppresses the hydrogen bond at positions P1, P2, P4, P6, and P9 (24) but appears to be compensated the others interactions at most of the positions. Rd (9, 14), Pp (17), and Cm had more drastic effects than Nm on binding to HLA class II molecules and T-cell recognition. To our knowledge, this is the first study to evaluate the impact of the introduction of Cm into a T-cell epitope on its immunological properties. Reduction of the peptide bond (Rd) removes the H-bonds at positions P-1, P2, P4, P7, P8 of the HA peptide (24) and disrupt the planar conformation of the peptide bonds. Rd, Pp, and Cm might indirectly reduce the binding capacity of the peptide by modifying its conformation (24). We also note that loss of T-cell activation is not necessarily a consequence of binding alterations, as exemplified by Pp introduced at positions 4, 5, and 6. These positions are in the central part of the peptide, the P5 position pointing to the TCR and the P4 and P6 being accommodated in corresponding HLA pockets. Pp locally disturbs the peptide conformation and the surface in contact with the TCR, which is sensitive to very subtle structural alterations of the interface (25).

This study also provides insights into the use of non-natural amino acids to minimize peptide- specific T-cell activation and the immunogenicity of therapeutic peptides. The risk of immunogenicity can be evaluated by introducing the sequence of natural amino acids in the therapeutic peptide into prediction software (IEDB, NETMHC, and Sturniolo) (12, 41, 42), thus providing an estimate of the binding IC_50_ for the selected HLA-DR allotypes and a predicted mode of peptide binding to HLA-DR molecules. By positioning the chemical modifications in the sequence with respect to the anchor residues, the impact on the binding can be deduced from the binding data presented in this paper by assuming that the modifications will have the same effect on the binding as we observed. Very few substitutions increase binding to HLA-DR molecules whereas, as we already stated, multiple substitutions especially of Aib and Daa promote a strong loss of binding. As shown in Figure 5, an ~3-log loss of binding completely abolished the capacity of the HA peptide to prime the T cells in the long-term T-cell assay. The cut-off required to abrogate T-cell priming by an immunogenic peptide appears to be high and might be higher than the threshold used to identify T-cell epitopes in a protein sequence. It might be especially high for strong peptide binders like HA (22). We also noted that introduction of non-natural modifications into a T-cell epitope is more effective at reducing HLA binding and hence T-cell stimulation when placed in the N terminal part of the peptide rather in the central and C terminal parts. The modification of P1-Y_308_ entails a sharp binding loss for almost all the non-natural modifications. Y_308_ is deeply accommodated in the P1 pocket of HLA-DR (43) and constitutes the main anchor residue of the HA peptide (14, 44). Substitution of all its amino acids, except Y_308_, by an alanine leads to a peptide as active as the HA peptide in binding HLA-DR molecules (14, 44). P1-Y_308_ is also surrounded by multiple H-bonds between the peptide backbone and the HLA-DR molecule, which might be altered by the non-natural modifications as described above. The predominance of Y_308_ is shared by all the HLA-DR molecules to which the HA peptide binds, as they possess the same P1 pocket characterized by a glycine in position 86 (22). Other molecules harboring a V in position 86 generally use two major anchor residues and the binding to these molecules might be differently affected by introduction of non-natural modifications. We suspect that modification at position P4 for DRB1^*^15:01 (45) and DRB1^*^03:01 (46) and at position P6 for DRB1^*^13:01 (47) strongly diminishes peptide binding, but this was not demonstrated in this study. Further experiments are required to generate a large set of data with non-natural modifications to train learning algorithms of binding and to develop software suitable to identify potential T-cell epitopes in therapeutic peptides.

In conclusion, we have comprehensively analyzed the impact on T-cell activation of six non-natural modifications widely used to design therapeutic peptides. None of the modifications increased T-cell activation or generated strongly immunogenic peptides. In contrast, they mainly diminished binding to HLA molecules and hence T-cell stimulation. Our data provide important clues to the appropriate use of non-natural modifications to minimize the immunogenicity of therapeutic peptides.

## Data Availability Statement

The raw data supporting the conclusions of this article will be made available by the authors, without undue reservation.

## Ethics Statement

Ethical review and approval was not required for the study on human participants in accordance with the local legislation and institutional requirements. The patients/participants provided their written informed consent to participate in this study.

## Author Contributions

AA designed and performed the research, analyzed the data, and wrote the paper. SM, SI, OD, and CP designed the research and analyzed the data. BM designed the research, analyzed data, and wrote the paper. All authors contributed to the article and approved the submitted version.

## Conflict of Interest

AA, SM, SI, OD, and CP were employed by Sanofi. The remaining author declares that the research was conducted in the absence of any commercial or financial relationships that could be construed as a potential conflict of interest.
