# Supplementary material for: Introduction of Non-natural Amino Acids Into T-Cell Epitopes to Mitigate Peptide-Specific T-Cell Responses
Source: Front Immunol. 2021 Mar 11;12:637963. doi: 10.3389/fimmu.2021.637963 (PMC7991740; doi:10.3389/fimmu.2021.637963)
Supplement: Supplementary file 1 [file Table_1.DOCX]

| HA  analogs | HLA class II molecules | | | | |
| --- | --- | --- | --- | --- | --- |
|  | DRB1*0101 | DRB1*0401 | DRB1*0701 | DRB1*1101 | DRB5*0101 |
| HA | 0.9 | 37 | 29 | 20 | 8 |
| Daa1 | 0.6 | 32 | 17 | 9 | 13 |
| Daa2 | 2 | 46 | 2 000 | 390 | 60 |
| Daa3 | 6 400 | 80 000 | >100 000 | >100 000 | 9 500 |
| Daa4 | 8 000 | 80 000 | >100 000 | >100 000 | >10 000 |
| Daa5 | 3 400 | 70 000 | >100 000 | >100 000 | >10 000 |
| Daa6 | 3 | 3 000 | >100 000 | 30 000 | 4 000 |
| Daa7 | 120 | 2 000 | >100 000 | >100 000 | >10 000 |
| Daa8 | 33 | 7 500 | >100 000 | 30 000 | >10 000 |
| Daa9 | 16 | 1 000 | 1800 | 7 000 | 4 000 |
| Daa10 | 260 | 3 000 | >100 000 | 60 000 | 4 000 |
| Daa11 | 90 | 1 000 | 7 000 | 9 000 | 700 |
| Daa12 | 3 | 300 | 64 | 450 | 17 |
| Daa13 | 0.6 | 30 | 23 | 20 | 7 |
| Aib1 | 0.3 | 20 | 11 | 18 | 3 |
| Aib2 | 8 | 95 | 7 700 | 4 000 | 500 |
| Aib3 | 60 | >100 000 | 460 | >100 000 | >10 000 |
| Aib4 | 10 | 75 | 6 000 | 4 000 | 280 |
| Aib5 | 1 | 30 | 780 | 300 | 600 |
| Aib6 | 0.7 | 55 000 | 75 | 150 | 5 |
| Aib7 | 6.8 | 45 | 4 200 | 6 000 | 900 |
| Aib8 | 0.8 | 140 | 90 | 550 | 140 |
| Aib10 | 9 | 490 | 16 000 | 8 000 | 5 000 |
| Aib11 | 20 | 25 | 3 000 | 350 | 95 |
| Aib12 | 0.7 | 32 | 9 | 100 | 5 |
| Aib13 | 0.2 | 6 | 6 | 8 | 2 |
| Pep1 | 0.5 | 13 | 120 | 74 | 5 |
| Pep2 | 45 | 740 | 8 400 | 8 000 | 3 700 |
| Pep3 | 4 000 | >10 000 | >100 000 | >100 000 | 14 000 |
| Pep4 | 5 | 14 | 710 | 6 000 | 280 |
| Pep5 | 9 | 200 | 3 000 | 20 000 | 2 500 |
| Pep6 | 0.4 | 82 | 290 | 45 | 10 |
| Pep7 | 0.3 | 450 | 16 | 200 | 15 |
| Pep8 | 0.4 | 20 | 93 | 450 | 13 |
| Pep9 | 0.4 | 25 | 28 | 6 | 6 |
| Pep10 | 2 | 30 | 2 000 | 220 | 49 |
| Pep11 | 0.4 | 49 | 14 | 550 | 13 |
| Pep12 | 0.3 | 4 | 10 | 14 | 3 |

**Table S1. IC_50_ of HA peptide and analogs containing Daa, Aib and Pep modifications**

36 analogs containing Daa (D-amino acid), Aib (amino-isobutyric acid) or Pp (peptoid) were submitted to competitive ELISA specific for five HLA-DR molecules. Mean IC50 were expressed in nM and result from at least two independent experiments.
