# Supplementary material for: Introduction of Non-natural Amino Acids Into T-Cell Epitopes to Mitigate Peptide-Specific T-Cell Responses
Source: Front Immunol. 2021 Mar 11;12:637963. doi: 10.3389/fimmu.2021.637963 (PMC7991740; doi:10.3389/fimmu.2021.637963)
Supplement: Supplementary file 2 [file Table_2.DOCX]

| HA  analogs | HLA class II molecules | | | | |
| --- | --- | --- | --- | --- | --- |
|  | DRB1*0101 | DRB1*0401 | DRB1*0701 | DRB1*1101 | DRB5*0101 |
| HA | 0.9 | 37 | 29 | 20 | 8 |
| Nm1 | 0.7 | 16 | 17 | 30 | 3 |
| Nm2 | 28 | 450 | 2 000 | 6 000 | 849 |
| Nm3 | 2 000 | 60 000 | 9 500 | 60 000 | >10 000 |
| Nm4 | 1 | 64 | 450 | 500 | 49 |
| Nm5 | 2 | 2 500 | 1 000 | 5 000 | 790 |
| Nm6 | 0.6 | 49 | 53 | 49 | 9 |
| Nm7 | 0.8 | 49 | 49 | 35 | 4 |
| Nm8 | 0.3 | 17 | 23 | 20 | 0.7 |
| Nm9 | 0.3 | 30 | 17 | 5 | 6 |
| Nm10 | 2 | 100 | 450 | 890 | 200 |
| Nm12 | 0.2 | 9 | 14 | 19 | 2 |
| Cm1 | 0.3 | 14 | 10 | 20 | 4 |
| Cm2 | 14 | 100 | 2 000 | 5 000 | 390 |
| Cm3 | 3 | 55 | 890 | 750 | 100 |
| Cm4 | 2 | 63 | 450 | 300 | 710 |
| Cm5 | 14 | 140 | 3 000 | 20 000 | 350 |
| Cm6 | 3 | 390 | 630 | 950 | 45 |
| Cm7 | 20 | 2 500 | 3 000 | 5 000 | 2 000 |
| Cm8 | 7 | 520 | 390 | 7 000 | 35 |
| Rd1 | 2 | 25 | 140 | 2 500 | 120 |
| Rd2 | 79 | 750 | 10 000 | 100 000 | 1 000 |
| Rd3 | 55 | 690 | 6 000 | 4500 | 3 000 |
| Rd4 | 600 | 8 000 | 20 000 | 6 300 | 6 500 |
| Rd5 | 9 | 50 | 1 400 | 5 500 | 200 |
| Rd6 | 250 | >100 000 | 7 000 | 4 000 | 3 500 |
| Rd7 | 1 | 200 | 1 400 | 290 | 50 |
| Rd8 | 2 | 200 | 1 300 | 840 | 95 |
| Rd9 | 45 | 650 | 7 300 | 3 000 | 140 |
| Rd10 | 250 | 4 000 | 7100 | 2 000 | 2 000 |
| Rd11 | 3 | 390 | 370 | 4 000 | 12 |
| Rd12 | 0.5 | 100 | 380 | 200 | 5 |
| Rd13 | 0.8 | 6 500 | 25 | 780 | 8 |

**Table S2. IC_50_ of HA peptide and analogs containing Nm, Cm and Rd modifications**

33 analogs containing Nm (N-methylated), Cm (C_α_-methylated) or Rd (reduced amide bond) were submitted to competitive ELISA specific for five HLA class II molecules. Mean IC50 were expressed in nM and result from at least two independent experiments.
